# Juvenile polyposis syndrome

**DOI:** 10.4103/0971-9261.44762

**Published:** 2008

**Authors:** Vijai D. Upadhyaya, A. N. Gangopadhyaya, S. P. Sharma, S. C. Gopal, D. K. Gupta, Vijayendra Kumar

**Affiliations:** Department of Pediatric Surgery, IMS, BHU, Varanasi, India

**Keywords:** Ileal pouch, juvenile polyposis syndrome, prophylactic surgery

## Abstract

**Aim:**

Report of a series of 12 cases of juvenile polyposis coli.

**Methods:**

The study period was from 1995 to 2005. All the patients were treated by total colectomy with rectal mucosectomy and endorectal ileoanal pullthrough with or without ileal pouch formation. Covering ileostomy was avoided in all the cases. Time taken for the surgery, postoperative complications and continence were documented.

**Results:**

The mean operating time was 4.2 h (range: 4–5 h). The mean duration of hospital stay was 16.3 days (range: 15–18 days). The most common postoperative complication was pouchitis and perianal excoriation. Initially, all the patients were passing stools at an interval of 2 h, and after 3 weeks, the frequency has reduced to 6–8 stools per day. In the follow-up after 3 months, the frequency was 3–5 per day with minimal soiling.

**Conclusions:**

Single-stage total colectomy with rectal mucosectomy and endorectal ileoanal pull-through without covering ileostomy and pouch formation is a safe and definitive treatment for juvenile polyposis coli if the patient selection is appropriate.

## INTRODUCTION

Juvenile polyposis coli (JPC) is one of the distinct entity of juvenile polyposis syndromes (JPS); others are diffuse juvenile polyposis of infancy and diffuse juvenile polyposis.[1] JPC usually occurs in children aged between 5–15 years. Patients with JPS present with bleeding per rectum, anemia or rectal prolapse; in JPS, polyps are usually limited to the distal colon and rectum[2] and about 50% of these patients have positive family history.[3] The patient with positive family history poses high risk for carcinoma (17%) occurring at an early stage,[3–7] or there is severe dysplasia in some cases.[8] These patients had cumulative risk of malignancy in 68% cases by the age of 60 years.[5] The purpose of this study is to review clinical and pathological features in JPS and its management with single-stage surgery and compare the two surgical procedures for its management (group B without pouch and group A with ileal pouch).

## PATIENTS AND METHODS

This is a prospective as well as retrospective study carried out from 1995 to 2005. All patients of JPS admitted during January 1995 to December 1999 were placed in (control group – 5 cases) group A, where as patients of JPS admitted during January 2000 to January 2005 were placed in group B (study group–7 cases). All patients underwent histological confirmation of polyps and then subjected to upper and lower gastrointestinal endoscopy and double contrast barium examination. Patients in group A were treated with single stage total colectomy and rectal mucosectomy with endorectal ileal pouch-anal anastomosis without covering ileostomy. The patients in group B were treated with single stage total colectomy and rectal mucosectomy with direct endorectal ileoanal anastomosis without covering ileostomy. The mean length of rectal muscular cuff was 6.1 cm (range: 5 to 8 cm). Hand-sewn ileoanal anastomosis was at about 1.5 cm to 2 cm from the dentate line. The outcomes of the 2 groups (A and B) were assessed on the basis of intra-operative time, duration of hospital stay, frequency of stools and anal continence (Kelly's scoring system). The study was permitted by the ethical committee of our institute and an informed consent was obtained. Statistical analysis was done by using Fisher exact test and Chi square test.

## RESULTS

A total of 12 patients were included in our study, out of which 9 were male and 3 were female. Mean age of presentation was 6.3 years (5 to 14 years). The distribution of polyps, adenomatous changes and presenting symptoms are summarized in Table 1. In the present study, 5 patients had positive family history and 3 patients had associated malformation in form of ventricular septal defect (VSD) and hypospadias. The most common presenting symptom was bleeding per rectum (83%), followed by anemia (66%), extrusion of polyp per rectum, rectal prolapse (8.3%) and protein-losing enteropathy in 8.3% cases. All patients underwent excision of polyp and in 11 patients; total colectomy with rectal mucosectomy and endorectal ileal pullthrough with ileoanal anastomosis without covering ileostomy was performed. One patient refused definitive surgery and was lost in follow up. In our series, we have operated 11 patients; in 5 out of 11 patients ileoanal-pouch anastomosis was done (group A), whereas in 6 patients direct ileoanal anastomosis was done (group B) [Table 2]. The mean operative time for group A was 5.1 h, where as it was only 4.1 h in group B. Mean duration of hospital stay was 14.2 (range: 12–16 days) in group A and 18.3 days (range: 16–20 days) in group B. Average frequency of stool after 3 weeks of surgery was 4-6/day in group A, whereas in group B, it was 6–8/day [Table 2]. Perianal excoriation was present in 20% cases of group A and 28% cases of group B. In follow up, patients of group A were passing stool 3–4 times a day, while patients of group B were passing stool 3–6 times a day without perianal excoriation (after 6 months of surgery) with fairly good continence. Three patients of group A were readmitted for fever, abdominal pain, and diarrhea (because of pouchitis) and one of them required pouch excision, whereas none in group B had such problemss. Anal continence was good, fair and poor in 60%, 20%, 20%, and 60%, 40%, 0 in groups A and B, respectively. Over all, complications were higher in group A in comparison to group B, but the difference was not found to be statistically significant. All patients were regularly followed up and doing well except the patient who refused to surgery. There was no mortality in present series.

**Table 1:** Site of polyps, precancerous changes and clinical presentation

Patients	Site of polyps	Adenomatous changes	Presentation
1	C,R,G		BPR, Anemia,
2	C,R	Present	Anemia, RP
3	C,R		BPR
4	C,R	Present	BPR, Anemia, Enteropathy
5	C,R		BPR, RP
6	C,R		BPR, Anemia
7	C,R	Present	BPR, RP
8	C,R		Anemia, RP
9	C,R		BPR, Anemia
10	C,R	Present	BPR, RP
11	C,R		BPR, Anemia, RP
12	C,R,G		BPR, Anemia, Enteropathy*

BPR : Bleeding per rectum, RP : rectal prolapse or extrusion of polyp through rectum

* Lost in follow up.

C = colon, R = rectum, G = gastric

**Table 2:** Type of surgery and postoperative observations

Patients	Type of operation	Per operative time	Hospital stay in days	Stool frequency at 2 weeks	Stool frequency after 6 months
**Group A**					
1	P, TC + RM + ERIAA + PC	330 min	17	8–9	3–4
2	P, TC + RM + ERIAA + PC	310 min	16	5–6	2–3
3	P, TC + RM + ERIAA + PC	320 min	14	5–6	2–3
4	P, TC + RM + ERIAA + PC	300 min	13	5	3–4
5	P, TC + RM + ERIAA + PC	330 min	15	6–7	2–4
**Group B**					
6	P, TC + RM + ERIAA	210 min	15	4–5	2–4
7	P, TC + RM + ERIAA	230 min	20	7–8	3–4
8	P, TC + RM + ERIAA	220 min	16	7	2–4
9	P, TC + RM + ERIAA	240 min	18	8–10	3–5
10	P, TC + RM + ERIAA	230 min	15	7–8	2–4
11	P, TC + RM + ERIAA	250 min	21	7–10	3–5
12*	P		2		

P : Polypectomy either manual or endoscopically

TC + RM + ERIAA + PC : Total colectomy with rectal mucosectomy with endorectal ileoanal anastomosis with pouch formation.

TC + RM + ERIAA : Total colectomy with rectal mucosectomy with endorectal ileoanal anastomosis without pouch formation

TC + RM + ERIAA + GP : Total colectomy with rectal mucosectomy with endorectal ileoanal anastomosis without pouch formation with gastric polypectomy.

* Lost in follow up

## DISCUSSION

JPS was first described by Mc-Coll in 1964. The etiology of JPS remains elusive, thought to arise as a result of allergic phenomenon[9] or considered as hamartomas.[10] Juvenile polyposis syndrome is generally characterized by multiple hamartomatous polyps throughout the gastrointestinal tract. In the present series, positive family history was found in 33.3% cases, which is slightly less than other reported series.[4,11,12] Presence of other associated anomalies was observed in 20% cases, which is similar to other reported series.[13,14] The diagnostic criteria for juvenile polyposis syndrome are somewhat controversial, but we have followed the guidelines outlined by Giardiello *et al*.[15] Initially, the indications of surgical intervention for JPS were as follows: when polyps are numerous and difficult to control endoscopically, if symptoms such as bleeding and diarrhea are troublesome, or when there is any suspicion of cancer;[16,17] however, recently, prophylactic surgical intervention is considered as the treatment of choice.

Presence of multiple juvenile polyps in JPS is associated with an increased risk of gastrointestinal malignancy,[7,18] with a cumulative life-time risk of up to 50%. Cancers most commonly occur in the colon, rectum and stomach,[7] although duodenal and pancreatic cancers have also been reported. Desai *et al*.[19] estimated that the projected incidence of colorectal cancer alone by the age of 60 was approximately 68%, and because of the risk of malignancy, these patients need surgery at early age.

Colectomy with ileorectal anastomosis or proctocolectomy with pouch creation[16,20,21] is the option available for patients with JPS. Traditionally, patients with JPS were treated with colectomy and ileorectal anastomosis, but due to high rate of recurrence, total proctocolectomy is now the treatment of choice. At our centre, we treat these patients with single stage total colectomy with rectal mucosectomy with endorectal ileoanal anastomosis without covering ileostomy, with or without ileal pouch formation. All the patients operated during 1995 to 2000 had ileal pouch-anal (S pouch) anastomosis. Since most of the patients 3/5 (60%) with pouch-anal anastomosis presented with feature of pouchitis in follow up and one of them required pouch excision, we opted for direct ileoanal anastomosis later on. Incidence of pouchitis was slightly higher in our series than other reported series[22–24] in pediatric patients, where it varies from 16% to 29%. In the present series, re-operation and pouch excision was done in only one case (20%), which is similar to other reported series.[24] Stool frequency was slightly higher in group B at the first follow up (at 3 weeks), and it was almost similar in both groups after 6 months, which is similar to the other reported series.[25,26] The observation of Morgan *et al*.[25] were similar to our study, and they concluded that straight ileoanal anastomosis remains an appropriate alternative for children and adults with ulcerative colitis or familial polyposis and compares favorably with the more complicated endorectal pull through involving a reservoir. In the present series, none of the patients with straight pull-through required conversion to pouch, which is similar to the results of Coran,[26] but contrary to the results of Fonkalsrud *et al*.[24] where conversion was required in about 90% cases. The perianal excoriation was slightly higher in group B, but the difference was not found to be statistically significant. These data show that the pouch-anal anastomosis is not advantageous in comparison to direct ileoanal anastomosis in children and is similar to other reported series. These results suggest that total colectomy with rectal mucosectomy with endorectal ileal pull-through with ileoanal anastomosis without pouch formation is a good alternative for ileal pouch anal anastomosis and can be performed in single sitting without covering ileostomy if the patient selection is appropriate.

For patients of JPS who had undergone surgical resection of bowel: endoscopic follow-up is required regardless of the surgical procedure because of the high rate of subsequent development of polyps in the rectum and the pouch.[21,27] Family members of patients with JPS should be screened[19,28,29] by the age of 15 years. If results are negative, upper and lower endoscopy is repeated every 3 years[30] until the age of 40 years. If results are positive, the patient should return for yearly upper or lower endoscopy until free of polyps.[31] Small numbers of polyps can be managed with polypectomy, but multiple polyps often require surgical management.

## CONCLUSION

Juvenile polyposis syndrome is an uncommon disease with definite malignant potential and need prophylactic surgery with regular follow up of patients and active surveillance for family members. We suggest that prophylactic total colectomy with rectal mucosectomy with endorectal ileoanal anastomosis without pouch creation and covering ileostomy is a good alternative for patients with JPS if the patient selection is appropriate. Endoscopic follow-up after both procedures is necessary because of high recurrence rates of juvenile polyps in the remnant rectum or pouch.
